# A Sorrow Shared is a Sorrow Halved: The Search for Empathetic Understanding of Family Members of a Person with Early-Onset Dementia

**DOI:** 10.1007/s11013-017-9549-4

**Published:** 2017-08-04

**Authors:** Silke Hoppe

**Affiliations:** Centre for Social Science and Global Health, University of Amsterdam, Nieuwe Achtergracht 166, 1018 WV Amsterdam, The Netherlands

**Keywords:** Empathy, Early-onset dementia, Family, Suffering, Shared experiences, The Netherlands

## Abstract

In this article, I explore how family members of a person with early-onset dementia in the Netherlands attempt to achieve empathetic understanding from significant others, and the barriers they encounter in the process. Based on qualitative interviews, I show that the type of relationship shapes the choices people have to communicate their suffering and their expectations regarding the reactions of others. This article builds on theoretical work on empathy and problematises the notion of shared experiences. It focuses on empathy between family members and significant others, arguing that empathetic understanding between these people is a field of study thus far insufficiently explored.

## Introduction

‘A sorrow shared is a sorrow halved’ is a common proverb in Western Europe. Being able to share one’s suffering and receive understanding makes the suffering more bearable and alleviates distress (Kirmayer 2008:460). If sorrows cannot be shared or are delegitimised, suffering increases (see Ware 1992). In this article, I explore how family members of a person with early-onset dementia seek empathetic understanding from significant others, and the barriers they encounter in the process. I demonstrate the complexity of the matter of empathy by problematising the role of shared experiences. Furthermore, I discuss dilemmas related to empathy and the role of emotions in reaching empathetic understanding. This article contributes to a better understanding of empathy between family members and significant others. While empathy has received a lot of attention in the professional domain, empathy in the private domain has thus far been insufficiently explored.

Several scholars argue that suffering cannot be expressed in words (Charmaz 2002) because it is incommunicable, and can only be hidden in general or metaphoric use of language (Das 1997; Morris 1997). Frank defines suffering as the experience of being “on the other side of life” (Frank 2001:355).

Suffering is the unspeakable, as opposed to what can be spoken; it is what remains concealed, impossible to reveal; it remains in darkness, eluding illumination; and it is dread, beyond what is tangible even if hurtful. […] Suffering resists definition because it is the reality of what is not (ibid.).

While it would seem that suffering has a quality that makes it inherently not shareable, De Wolfe (2002) has criticised this view, arguing that it increases the isolation and stigmatisation of the suffering person. She emphasises that people living with illness need social recognition and support, which cannot be achieved if they keep their experiences to themselves. Instead of viewing suffering as something that cannot be shared, we should analyse how and under which conditions it can be shared. Kirmayer reminds us that in order to express suffering, people need a “receptive audience” (1996:193).

But what characterises a receptive audience? One important feature is empathy, which “reflects the willingness to meet, engage, and be moved by the other” (Kirmayer 2008:458). Scholars disagree on whether empathy is an intellectual construction that requires cognitive skills or an affective ability that depends on emotional resonance (see Preusche and Lamm 2015). According to Halpern (2003), within the field of medicine, empathy is considered a form of detached cognition, while outside of medicine it is seen as an affective mode of understanding. In her book *From Detached Concern to Empathy*: *Humanizing Medical Practice*, Halpern (2001) argues that only a combination of emotional resonance and cognitive imagination can allow for empathy. Resonance, which is non-cognitive, is insufficient. “Resonance is like stage lighting or music in that it can attune people to common themes, but it cannot magically and silently present to those involved a common, complex emotional viewpoint” (Halpern 2001:80). Preusche and Lamm (2015) support this notion by stating that theoretical and empirical evidence underlines the affective nature of empathy, demonstrating that the view of empathy as mainly cognitive is outdated. Instead, they argue that empathy has both affective and cognitive elements.

Even though scholars differ in terms of how they conceptualise empathy, all agree that empathy does not happen automatically but requires work.

One cannot just rely on one’s own store of memories, images, and experiences to imagine the plight of another, especially from another society or culture. Rather, one must, through hard work and effort, gain access to their symbols and meaning (Geertz) or positionality (Rosaldo) or compelling concerns (Wikan) in life (Hollan 2008:480).

Although most attention is focused on the empathiser, empathy demands effort on both sides (Hollan 2008). On the one hand, the empathiser needs “a willingness to engage with another world, life, or idea” (Wikan 1992:463); but on the other hand, the person one wants to empathise with has to give the right clues for understanding to be possible and at least make an effort to imagine to be understood. This process requires time (see Throop 2010). Only through constant dialogue, in which experiences and interpretations are confirmed or disconfirmed, can understanding and empathy be achieved (Halpern 2001:88; Hollan 2008:476; Wikan 1992:468). If a person does not want to be understood, does not give the right clues or does not make enough effort to feel understood, empathy will fail (see Hollan 2008). Refusing to be understood can be a way of creating a sense of exclusivity and thereby be a means to gain power.

Although scholars probably agree that time, work and dialogue are necessary for empathy, they disagree when it comes to the role of shared experiences. Fainzang (2007:11) discusses two positions with regard to the role of shared experiences in empathy. She states that while some scholars argue that people can only experience empathy if they have had an identical experience, other scholars see empathy as an intellectual construction that can be reached even if one has not had similar experiences. For Rosaldo, for instance, similar experiences are crucial for understanding the other. Only after his wife had died in an accident could he grasp the grief and rage of the people he studied (Rosaldo 1989:9, in Hollan 2008:478). Fainzang (2007:11) states, however, that although empathy involves an emotional aspect, it is nevertheless an intellectual construction; one can empathise with people who are in a situation that one has not personally experienced. An anthropologist, for example, who has not experienced alcoholism can still imagine and reconstruct this experience (ibid.). Wikan (1992) agrees, arguing that it is not necessary to have had the same experiences in order to understand the other; instead, it is important to be exposed to the other’s world and to participate in it on a regular basis.

When discussing the role of shared experiences in empathy, it is important to first problematise the notion. What exactly counts as shared experience? And are shared experiences always helpful in understanding the other? Kirmayer (2008) emphasises that the duration and intensity of an experience is crucial; someone who has experienced headaches cannot necessarily understand the experience of chronic headaches. He further explains that empathy may fail if there is a mismatch in the experience. Thus even though two people might have the same illness, their age, gender, ethnicity, biography, education and socio-economic background will still strongly shape their experiences. An Asian middle class man in his sixties might experience chronic headaches very differently from a European working class woman in her twenties.

Furthermore, what qualifies as a match or mismatch in experience may differ between the empathiser and the person who wants to feel understood. A person who has experienced headaches might feel that he or she can perfectly understand what it means to have chronic headaches, while the person suffering from chronic headaches might feel that the other person has an incorrect understanding. This also raises questions about power, about who can afford to make a flawed estimation of the other’s situation and who depends on a correct assessment (see Povinelli 2001; Wikan 1992).

There is also a danger that projection may be confused with empathy. According to Geertz (1984, in Hollan and Throop 2008:388), anthropologists have too often projected their own thoughts and feelings onto others and have thereby assumed an incorrect understanding. Similarly, Halpern (2001:83) warns that by confusing projection with empathy, important differences in experience are neglected. Thus Throop (2010) points out that while similar experiences in certain situations may make empathy possible, in other situations they might impede it.

In her book *The Pastoral Clinic*, which deals with heroin addiction in New Mexico, Garcia (2010) explores the concept of commensurability and thereby paints a more complex picture of shared experiences. She demonstrates that sharing an experience does not necessarily lead to connection and understanding: “Although patients may collectively feel pain, they do not share it. The pain forms a kind of inconsolable solitude” (Garcia 2010:50). Regardless of whether people have the same affliction and background, the pain they all experience does not necessarily unite them. Moreover, Garcia reflects on her role as a researcher and the limits of empathy. She writes that when she spent a night with a patient, she was unable to fully understand the nature of his pain and needs. Although she experienced a connection and a common vulnerability by staying at his side, she was unable to grasp his suffering. Thus it is crucial to acknowledge the complexity of the issue of shared experiences in empathy. The relationship between the two is not straightforward, and the question of which experiences are *not* shared is just as important as asking about the experiences that are.

Another important aspect in empathetic understanding is language: “People often cannot say what they mean or feel, either because they don’t know or because it would be inappropriate. Many things are left unspoken or are self-evident to speakers” (Hollan 2008:478). People might not want to come across as complaining or assume too much knowledge of the other. As a consequence, if suffering is not explicitly expressed, it can be hard to recognise.

Although much has been written on empathy in anthropology, a lot of work focuses on the position of the anthropologist (Briggs 2008; Fainzang 2007; Garcia 2010; Wikan 1992) or the relationship between doctors and patients (Halpern 2001; Kirmayer 2008). Much less has been written about empathy between family members and significant others. For example, Halpern (2001, 2003), who focuses on clinical empathy, seems to assume that outside the field of medicine, empathy is an affective mode of understanding. Yet there is little research that actually describes empathy in a private context. This article shows that reaching empathetic understanding is achieved in different ways depending on the relationship between people.

Studies on caregivers mostly focus on social support or recognition, rather than on empathy. Although it is acknowledged that social support and recognition have a positive effect on caregivers (see Chappell and Funk 2011; Chen and Greenberg 2004; Zhan 2006), how this support and recognition is received is not problematised (see Carbonneau et al. 2010). Even though Ribeiro et al. (2007) state that their study participants—older men caring for their wives—did not have the impression that their care work was invisible or unacknowledged, many other researchers, including Russell (2001), have found this to be the case:

Many felt that others, especially family members, did not appreciate the extent to which their entire lives were bound up in the demands of caregiving. They also described how others claimed to understand their situations but, in reality, could not, because of only minimal and sporadic contact (Russell 2007:310).

Bjorgvinsdottir and Halldorsdottir (2014) describe how children caring for sick parents felt that their care work was invisible and unacknowledged. Based on the literature, the impression arises that people who are not typically associated with providing care—such as children or men—may have difficulties in receiving recognition and understanding. In this article, however, I argue that even those who are typically associated with care—such as women—may also struggle to receive empathetic understanding. One explanation can be found in what Moore and Gillespie (2014) term the ‘caregiving bind’. Through the provision of care, the care that the sick person needs is paradoxically often concealed.

In this article, I use early-onset dementia as a case to demonstrate how family members, male and female, try to achieve understanding and recognition from significant others, and the dilemmas they encounter in the process. Although dementia has received significant attention in both public discussion and scientific research, this is less true for early-onset dementia, a diagnosis that people receive before the age of 65, and which occurs in about 3–5% of all dementia patients (Bakker et al. 2010:634). In contrast to late-onset dementia, early-onset dementia comes in many different forms. While among late-onset dementia patients Alzheimer’s disease is the most common form of the illness, in early-onset dementia it only represents one-third of cases; other dementias include Lewy body disease, Pick’s disease, frontotemporal dementia, vascular dementia, and alcohol-related dementia (Beattie et al. 2004:359–360). Thus people with early-onset dementia often present with different symptoms than those commonly associated with Alzheimer’s disease. Memory loss, for instance, is not necessarily the most common symptom. They may rather display neuropsychiatric symptoms that become apparent in limited illness insight and changes in socio-emotional behaviour (van Vliet et al. 2010:1092), such as displaying less initiative, more compulsive behaviour, apathy and irritability.

As early-onset dementia is both a progressive illness that becomes increasingly visible and a rather unknown illness associated with stigma (Ballenger 2006; Kontos 2006; Milne 2010), it provides a good opportunity to explore the efforts that family members make to achieve understanding and recognition, the strategies they use to receive empathy from others, and the dilemmas they encounter in the process.

## Methods

This article is based on forty-one semi-structured interviews that I conducted among people with early-onset dementia and their family members in the Netherlands. Research participants were recruited via the national Alzheimer’s Society and three care institutions specialised in early-onset dementia. Over a period of 1 year, I interviewed three people with early-onset dementia individually (one of them twice); four persons with early-onset dementia together with their partner (in one case children also participated); and 41 family members. In total, 11 group constellations were interviewed, consisting of the person with early-onset dementia and his or her partner, a partner and a child, siblings, or a whole family. The majority of the interviews took place in people’s homes, though two were conducted in my office and three in a public space such as a café. The majority of those interviewed lived in large cities, while some lived in small towns and a few in rural areas.

The youngest child of a parent with early-onset dementia was 19, the oldest 43; the youngest partner I interviewed was 47, the oldest 67. The age of people with dementia ranged from 55 to 65. The interviews lasted between 1 and 5 h, with an average of 2 h. In the interviews, I asked people to describe the period from when they first noticed that something was wrong up to the present. I addressed the impact of the illness on their lives and relationships and asked how they dealt with the changes caused by the illness. In addition, I asked how people talked about the illness, with whom they talked, and how people reacted to their stories. The interviews were recorded and subsequently transcribed. I coded the interviews using the qualitative data analysis software NVivo, and based on the codes I re-read several of the interview transcripts in order to decide which three cases to present in this article. The issues discussed below arose in the majority of the interviews. Although this article addresses interactions between family members and significant others, I only interviewed family members and thus everything stated in this article is based on their perspectives.

Ethical approval was granted by the Amsterdam Institute of Social Science (AISSR). All participants were informed about the aims and objectives of the research. Verbal informed consent was obtained from all individual participants included in the study. To assure anonymity, pseudonyms are used for all participating informants, and in some cases identifying details have been changed.

## Thamara and Franka

Thamara reacted to my call for research participants on the website of the national Alzheimer’s Society. At the time of the interview she was 31 years old, the daughter of Franka (62 years) and Klaas (65 years). I first interviewed Thamara at her house in a suburb of a large city together with her mother for about 3 h, and a few months later I interviewed her alone for 2 h. When Thamara was in her twenties and no longer living at home, the family started to notice that little things with Klaas were not quite right. Although he had always been a very active person, he began sleeping on the couch after returning home from work and he expressed the desire to stop working. When driving, Franka noticed that he forgot to turn off the indicator, and Thamara realised that he often went to the kitchen to get something but would return with nothing. In the beginning, Franka suspected that her husband was having a burnout. But 8 years prior to our interview, after several tests at the hospital, Klaas, at the age of 57, was diagnosed with early-onset dementia.

After receiving the diagnosis, Klaas was declared unfit for work and stayed at home. Franka, who had previously done voluntary work, cared for him. As Klaas was physically still very active, he insisted on biking every day, regardless of the weather. But one time, after he had gotten lost while biking to Thamara’s sister’s house, Franka decided that he was no longer able to bike by himself and would accompany him. When Franka noticed that he was crossing roads without checking for cars, they switched to taking walks together. A few years later, around Easter, Klaas developed a lung infection and needed to be hospitalised. In the hospital, Franka realised that he could no longer live at home and, with difficulty, they found him a place in a nursing home.

In the first years after the diagnosis, Klaas’ illness was hardly visible to outsiders.

Franka: We walked through the village and people said, ‘He looks good, he looks good’.

Thamara: My mother cared very well for him. He always went in a suit and went to the hairdresser and wore nice clothes. He ate messily, but my mother always took care of that.

Franka: Of course, you didn’t see that anything was wrong with him.

Later, in the second interview, Thamara reflected:

Yes, my mother took care of him so well and he always looked good. So people told my mother not to make such a fuss. They had no clue what my mother had to do for him and what she went through. They just saw a man who looked good. That was a very difficult period.

Although people knew that Klaas was sick, both Thamara and Franka suggested that others underestimated the situation. Paradoxically, the hard work that Franka put into caring for her husband made her work and care invisible.

As discussed earlier, people can only empathise with someone if they receive the right clues. In the case of Klaas, Franka and Thamara, one might say that at the beginning of Klaas’ illness, people did not receive the right clues because Franka hid them and thereby excluded the possibility for her to receive understanding from others. Although I did not explicitly ask why she had done so, based on my overall impression from the interview I would say that Franka saw it as her duty to provide good care for her husband, and probably she also wanted to maintain the good reputation he had in the village. In the end, it was Thamara who recognised how difficult the situation was for her mother. Franka did not complain. Possibly, she took pride in the fact that no one could see how much help Klaas needed. For her, protecting his identity was more important than for her daughter, who wanted to feel understood, as the following fragment shows.

When I interviewed her, Thamara was working as an occupational therapist at a psycho-geriatric ward, which meant that her colleagues were regularly working with people with dementia.

Thamara: At my work, my colleagues were very understanding, they all know what it means. And my friends… well it’s too much. If you say, ‘My father has cancer’, everybody has a picture in his head.

Franka: With dementia people don’t have this picture in their heads…

Thamara: You see that people don’t know how to react.

Even though it may be debatable whether an outsider will necessarily know what it means to have cancer, the seriousness of the illness is immediately established. Although Thamara at first pointed out that her friends did not know what it is like to have dementia, her last sentence rather points to a problem in the interaction, namely that people do not know what would be an appropriate reaction.

In the first interview, it was mostly Franka who talked about the care that her husband needed. Although Thamara pointed out from time to time how difficult the situation was, her mother simply continued to describe the practical challenges and avoided talking about the emotional consequences. After the interview, when Thamara drove me to the station, she seized the opportunity to communicate to me how much they suffer. She told me that when her father had had a hip operation some months before and again had to stay at the hospital, she had stood next to his bed and pushed a pillow onto his face, thinking to release him from his suffering. After a few seconds she had removed the pillow, wondering what she was doing. The image she depicted had a clear impact on me. It summed up very succinctly the suffering that Thamara experienced and that her mother did not seem to want to acknowledge. By telling this story, Thamara made sure that I, the interviewer, would assess the situation correctly.

When I interviewed Thamara alone for the second time, I returned to the situation with the pillow. She retold the story, but this time simply stated that she had only considered pushing the pillow against her father’s face. I inquired whether she had told her friends about it.

I told it to my friends, but they didn’t really react to it. I told them, but that didn’t lead to a lengthy conversation. I told it to show them how heavy and emotional it is and how bad he was. And crazily, it’s almost unethical, or that’s what I think, I took pictures with my mobile phone, how he looked. And then I tried to explain it with words, but people cannot have sympathy with that. And then I showed them the picture, and it happened twice that friends burst into tears. It was then that they got it. That’s why I did the photography course, because I believe that pictures sometimes say more than a thousand words.

Although Thamara had tried to explain the situation to her friends, she had not got the reaction she desired. This can be interpreted in different ways. Possibly, by simply describing the situation, Thamara had not provided the clues her friends needed to really understand the situation. Another interpretation is that her friends intellectually understood Thamara, but because they did not show an emotional reaction, Thamara was not sure whether they had really understood it. Maybe Thamara needed an emotional response in order to be content and to feel understood. This points to a desire for different responses from different audiences. With her colleagues, Thamara was satisfied with their intellectual understanding of the situation; but from her friends, she needed an emotional reaction. In contrast to what she had stated earlier, when she took the pictures of her father in the hospital, she did so in order to communicate the suffering of her father and what it did to her, and not so much to explain what early-onset dementia entails. The fact that she felt slightly guilty about taking the pictures of her father illustrates that, like her mother, she was concerned with protecting her father’s dignity. Thamara’s case thus illustrates not only how important she considered it to be understood and the lengths she would go to in order to reach this understanding, but also that different responses are expected from different audiences at different times.

## Ellen and Lianne

When I met Ellen (55) she was married to Nico (61), and together they had three children aged 24, 26 and 29. I interviewed Ellen twice together with her middle child Lianne (26) at my office. The first interview lasted 2 h and the second an hour and a half. Two years previously, Nico had been diagnosed with early-onset dementia, but 4 years before the diagnosis his family had already suspected that something was wrong. For example, they had observed that Nico had started to withdraw from conversations and had become absent. At first, they had thought this was due to a hearing problem, but after he had received a hearing aid nothing changed. When Ellen’s mother had died of Alzheimer’s disease and Ellen had informed Nico over the telephone, he had simply said ‘Oh, oh’, and then passed the receiver to his daughter. He also took the wrong exits when driving. As Nico had not wanted to see a doctor, it had taken a long time until he had finally received a diagnosis.

In the following quote, Ellen established how difficult the situation was for her.

You do not get the sleep you need, and as a consequence you are dead tired. And then you end up in a circle you cannot escape, you are on your last legs and then you get a period, a few days that you are really desperate, and if someone says ‘Boo’ you start crying, and if someone is nice you also start crying. And then you recover… Last January I had quite a crisis. I just couldn’t handle it. And then I reacted not so [nicely]… I try for him, because it is pitiful, but at a certain moment it is also a kind of self-protection, I couldn’t handle it, thus what do I do then… [Ellen becomes emotional and cries]…I took a few days off and that helped me a lot. Because otherwise every two months I have a crisis. The next one is coming up. No… just kidding.

When Ellen cried, Lianne consoled her and emphasised that it is only natural that Ellen struggles. Yet not all family members were so understanding.

At the time of the interview, Ellen was working part-time. For quite some time, when she was not at home, Nico had merely slept on the couch, even though he was physically still quite active. They had therefore found him a place at a social care farm where he could engage in physical activities, which he considered his work. At first Nico had not wanted to go to the care farm, thus Ellen had found it difficult to send him there.

Ellen: I learn, but I also feel guilty. Also towards him. You think you put him away. Especially the first time when we went there, his sister… she made certain comments that she didn’t agree and that she didn’t understand why he had to go there. You already feel guilty that you send him there, but you wanna give it a try, because at home he didn’t do anything anymore. And she [Nico’s sister] says, ‘Yes, but he worked for years and years, let him enjoy his days’. Yes, but nothing comes out of it anymore.

Lianne: He merely sleeps if he is alone at home. He doesn’t do anything.

Ellen: He is very happy there. He really enjoys it, because everything is arranged and he doesn’t need to think about a thing […]. But if you already feel guilty and certain comments are made then it hits you hard. I had to learn to deal with that, and not to let it affect me. I just want confirmation, for example from his sister, that I am doing the right thing.

Even though Ellen believed that her choice was in her husband’s interest, she felt guilty about sending him there. His sister’s lack of understanding made the situation worse, because it fuelled rather than tempered her feelings of guilt.

The next fragment from Lianne illustrates how the absence of acknowledgement not only made the situation more difficult for her mother, but also caused feelings of anger.

What I notice is that sometimes it frustrates you [Ellen] very much that people don’t know what impact it has, that they say things like, but that are more acquaintances or neighbours, that they say, ‘Yes, he sometimes forgets things’. Then you boil with rage. Or my father’s sister, she just sees things differently, she finds it pitiable that on Saturday he also goes to the social care farm. But she doesn’t realise how difficult it is for my mother. When he is staying there for the night my mother says, ‘I hope that she will see how hard it is’. If he stays with his sister for a few days, she will see how difficult it is. I notice that you can hardly get understanding from people who are not in this situation. Because it is almost incomprehensible.

Ellen was happy to send Nico to his sister so that she could have a weekend off; but beyond this, she also hoped that his sister would get a better impression of the situation and possibly change her perspective. This can be seen as a way of trying to receive understanding from the sister. Instead of using more words to explain the situation, letting Nico stay at his sister’s place for a weekend could be interpreted as a strategy to create shared experiences.

Ellen realised that the situation was very difficult for outsiders to understand.

It is something very intangible, you cannot imagine. If, for example, someone talks to him on the street, and you would start talking about soccer, he has a season ticket for the local soccer club, so he would tell you about it. And nobody would realise that he has Alzheimer’s disease. Only if you are with him for a bit longer do you realise that things are not right and that he repeats stuff.

One could say that the clues that Ellen gave to others to communicate how much help Nico needed were being undermined by her husband’s appearance and behaviour. While Franka above hid the seriousness of her husband’s condition through her good care, Ellen talked about not being ashamed of the illness. She told her husband that he should not be ashamed of it and he confirmed that he was not. Thus hiding the symptoms of the illness seemed to be unimportant for Ellen. By contrast, she described in detail the kind of care that her husband needed in order to receive understanding.

Lately at my work I said, ‘I have to tell him that he needs to shower, I have to tell him that he needs to brush his teeth, I need to prepare his clothes’. Some time ago he put a t-shirt on under his normal t-shirt and over it a vest and another vest. Also with food… lately I bought eight apples and put them in a basket. My son ate one and my husband ate the other seven apples in one day. You know, those kind of things you don’t see from the outside. Thus I said, ‘I have to prepare his clothes, I have to tell him to shower and brush his teeth’. But you don’t see that from the outside. They don’t experience those kinds of things. But then people are startled: ‘Oh, is it that bad?’ Yes, it is that bad.

At first glance, it would seem as if Ellen wanted to communicate how much help her husband really needed. But behind these efforts might lie a wish for legitimation. Had her colleagues only acknowledged her responsibilities, Ellen might not have been content. What counted for her was the moral judgement that the situation was indeed ‘that bad’.

As with other cases, this case demonstrates that a diagnosis does not necessarily lead to understanding and recognition. The partial invisibility of her husband’s condition—the illness was visible to insiders, but not to outsiders—and people’s general lack of knowledge of early-onset dementia both led to a delegitimisation of Ellen’s experiences. Yet the case also illustrates that family members require a certain kind of reaction in order to feel understood. While on the surface the above quotes depict the fact that outsiders did not necessarily know what early-onset dementia entailed for the family, what Ellen seemed to be striving for was a confirmation that she was doing the right thing.

## Juliette and Hans

Juliette, at 19, was the youngest child of a parent with early-onset dementia that I interviewed. While her father Hans (52) was still at work, I first interviewed her alone for about 1 h. Then Hans joined our conversation and we talked for another hour, after which Juliette left and I talked to Hans alone. In total, I stayed in the family’s kitchen for 3.5 h. Juliette described how at the beginning, when things had started to become difficult with her mother Yin, she had thought that her mother was over-stressed with the care of her disabled son. Juliette had had many arguments with her mother, and when she was 9 years old she had been sent to a psychologist. Two years later, Yin was diagnosed with early-onset dementia at the age of 49. Yin had fits of rage and could hardly be left alone. She also suffered from apraxia, thus she no longer knew how to do basic things such as use kitchen utensils. The situation at home had been very difficult for Juliette and therefore twice, for a period of a few months, she had lived with the family of a friend until her mother had moved into a nursing home, where she was living at the time that I met the family. Hans felt guilty about the fact that by letting Yin live at home for so long, even though the situation had been unbearable for Juliette, he had chosen his wife over his daughter. For a long period he had been depressed and had had suicidal thoughts, which he discussed in the interview in the presence of his daughter.

Juliette found it difficult to talk about her mother with friends.

A lot of people say, ‘I understand how you feel, my grandfather or grandmother also has Alzheimer’s’. Then I think, ‘Yes, it’s your grandfather or grandmother, that’s a different story’. A lot of people also forget the transformation people go through. It’s not only forgetting, they become a different person.

Later she added:

Sometimes I say it is easier to explain to people that my mother is dead; they understand that: ‘Oh you don’t have a mother anymore’. But if I say my mother has dementia [they think]: ‘Oh yes, she forgets things, she is still there’. That is very different. Yes. Sometimes it is very annoying to explain.

Although people, mostly peers her own age, did try to relate to Juliette’s situation, she did not feel understood or could not imagine being understood. Different things seemed to be at stake. According to Juliette, most people associated dementia mainly with forgetfulness, and ignored the fact that it can change a person’s personality. Moreover, people did not seem to realise that for the majority of her life, Juliette had grown up without a mother. Although her mother was physically still there, already for many years she had been unable to fulfil the role of a mother.

For Juliette, it felt as if others merely projected their own ideas and experiences onto her situation, but failed to see the differences, which according to Juliette were very important. While her peers might miss a grandmother as much as Juliette missed her mother, she felt that her situation was different on many levels. Not only had it become natural for her to go to buy groceries and do the laundry from a young age, but she had also been accused of causing problems with her mother, and had grown up with a father who was depressed due to the circumstances and who had promised to put his children first but had twice chosen for his wife, something that had resulted in Juliette leaving home for periods of time to live elsewhere.

When Juliette tried to talk to her friends about something difficult regarding her mother, she complained that after 2 min they would simply make “stupid jokes” about something else, giving Juliette the feeling that they did not understand the gravity of the situation. Juliette emphasised that most people her age led very different lives and did not have to worry about the things that preoccupied her.

There are very few people with whom I have this conversation [about my mother] or want to have it. Then they say, ‘I understand you’, but they don’t! And that’s very annoying, that people say these kinds of things. I now have a boyfriend and his father died very young, when my boyfriend was seven. Thus he knows how it is to have one parent in the family, and his sister too, and his mother too. Thus in that sense I can talk with them easily. Because they know how it is to lose someone. It is very different, but it is still good to talk, because you are in a similar situation. It is nice, because you are in the same situation at home with one parent. Other people say, ‘Oh, you do your own laundry, you do that? You do the groceries?’ That kind of thing. Yes, my peers do not have to think about those kinds of things.

The above quote demonstrates that according to Juliette, a meaningful conversation about her mother could only happen with people who have had a similar experience. While her peers might know what the main symptoms of dementia are, according to Juliette they did not know the experience of losing a parent.

It seemed that there were many aspects of her situation that Juliette wanted to be understood: the symptoms of early-onset dementia, what it does to a person and to relationships with that person, and what it means to lose a parent. Interestingly, Juliette felt more understood by her boyfriend who had experienced the loss of parent through cancer than by her peers whose grandparents also had dementia. While Thamara above referred to cancer to illustrate how different it is from dementia—in the sense that unlike dementia, everybody knows what cancer entails—for Juliette, the fact that her boyfriend had lost a parent to cancer was in some regards comparable to her experience of having a parent with early-onset dementia. This comparison illustrates the complexity of reaching empathetic understanding. A family member of a person with early-onset dementia might say that others do not understand what the illness entails, but they might actually mean that others cannot grasp the impact that the illness has on the relationship.

Just like Ellen, Juliette wanted to let others experience how it was to have a mother such as hers.

Sometimes I have the tendency to tell people, ‘Just come and have a look’, then I will show you what kind of state my mother is in. This is something I really want to do. She will come to my graduation ceremony and I want to show my classmates: ‘Look at my mother, this is how she is now. And this maybe is the reason I had to redo this year’. This is something I would like to do, to confront people with the facts. Maybe that sounds bad. Because sometimes people just don’t show any respect, saying ‘Oh, you didn’t have a childhood, ha-ha’, that kind of thing.

At the end of our interview, I asked Juliette whether she would give me permission to use her story in my publications. She replied:

I hope that maybe a nice book will come out of it so that people maybe understand a bit more what it’s like, also with other stories. That people can understand, outsiders, what it is. What it does to you. I also told my father, maybe I want to join a TV programme, so that I can show people: that’s what it’s like, this is early-onset dementia, and that’s what it does to the family. It is very hard to tell, yes, to explain that to others.

This quote shows how strong Juliette’s desire was to be understood and the lengths she was willing to go to in order to achieve it. As in Thamara’s situation, Juliette suggested that images might perhaps be more powerful than words in conveying what it is like to live with a parent with early-onset dementia.

Juliette’s father Hans also pointed to the difficulty of explaining the situation to people who have not had similar experiences.

For some time I had sessions with a psychologist. But I really thought, ‘Do I have to explain each time what I experience?’ I didn’t have the feeling I achieved something more. Whereas at the partner meetings, you said a few words, but the others already understood you. Most had older partners, they were ten years older, but if you explained something, you thought, ‘At least they really understand what’s going on’. Without these conversations, I wouldn’t have made it, I know that for sure. Just talking with peers helps a lot. There are others who go through the same, who struggle with the same problems. I am not alone in this. They also struggle with other issues, but you get recognition and they can make a comment that helps you understand things better. You learn things through sharing experiences with others that you cannot put on paper.

In contrast to his daughter, Hans received a lot of support and understanding from people who were also caring for a partner with dementia, even if those people were much older than him, often no longer worked, and did not have children living at home. Although the psychologist he saw was trained to help people and listen empathetically, Hans did not feel that the therapy had helped him much. Instead, the common experience he had with the others in the support group—of being the partner of someone with dementia—was what helped him to achieve a sense of being understood.

This case shows that both father and daughter were searching for people with similar experiences by whom they could feel understood. For Hans, these were other partners caring for a person with dementia, while for Juliette, who had a different relation to the person with dementia than her age-peers, it was a family who had experienced loss as a result of cancer.

## Discussion

The empirical data presented above shows that family members of people with early-onset dementia find it important to receive empathetic understanding from others. In the following section, I discuss how this understanding is reached and the kinds of stumbling blocks that people encounter in the process. In the Introduction, I discussed the role of shared experiences, and stated that it is important to first clarify what we mean by this. Even though at first glance the empirical data could create the impression that shared experiences are necessary for empathetic understanding, on closer examination it becomes apparent that the situation is more complex. For example, even though an external person may also have a family member with dementia, this does not necessarily lead to empathetic understanding. On the contrary, as in the case of Juliette, it may in fact estrange people. While Juliette’s father experienced empathetic understanding from older people with a partner with dementia, for Juliette the difference between late-onset and early-onset dementia, or between being a daughter or granddaughter of someone with dementia, was too big. For her, the crucial element of her experience with her mother seemed to be the loss of a parent. This was something that she shared with her boyfriend, who had lost his father to cancer, but not with her peers who had a grandparent with dementia. This illustrates how important it is not to assume that shared experiences will necessarily lead to greater understanding. Even though the empathiser might have good intentions and make an effort to understand the other, this does not automatically lead to mutual understanding. Indeed, in some cases an assumed shared experience may prevent real understanding.

A further complication is that living with a person with early-onset dementia is a complex experience, which has many layers. The empirical data suggests that people’s needs with regard to the experiences they wish to be understood differ. In some circumstances, knowledge about the specific symptoms of early-onset dementia might be desired, whereas in other circumstances what seems to be important is recognition of the severity of the condition or of its effect on people’s relationships. As in the case of Thamara, sometimes an emotional outburst might be sufficient to feel understood. Whether a person feels understood is thus not only a matter of providing the ‘right’ reaction, but also providing the ‘right’ reaction at the right time. And to make the situation even more complicated, depending on the relationship one reaction might be considered ‘right’ with one person and ‘wrong’ with another.

This points to the role of emotions in empathetic understanding. Understanding cognitively what the symptoms of early-onset dementia are and how they influence a person’s life might not be enough for a family member to feel understood. It seems that understanding the seriousness of the situation can best be communicated with an emotional reaction. Yet an emotional reaction is not required from everyone. Thamara felt understood by her colleagues, who probably did not cry; but she only had the feeling that her friends had ‘got it’ when they had an emotional outburst. Her friends would probably not be able to list all the symptoms of early-onset dementia, but they saw the suffering caused by the illness and it seems that this was what had counted for Thamara at that moment. Thus different reactions are expected of different people.

Halpern (2001) argues that emotional resonance is insufficient for grasping another person’s distinct emotional point of view and achieving empathy. In some situations, however, feeling emotionally understood seems to be what people long for. It is possible that pain may be more easily shared through an emotional connection, where it is less relevant that people completely understand one another’s feelings, motivations and thoughts.

One strategy to receive empathetic understanding is to let people have similar experiences. Instead of additional explaining, family members attempt to show others what it is like to live with a family member who has early-onset dementia. This relates to Wikan’s notion of building empathetic understanding through participating in another’s world: “Sharing a world with others means learning to attend to it in the same way” (Wikan 1992:471). Even though, according to Wikan, shared experiences are not necessary for understanding the other, making a person participate in your life is one way to create some kind of shared experiences. As Garcia (2010) has demonstrated, sharing an experience does not necessarily lead to connection, but for my interviewees it seemed helpful in reaching understanding.

Another strategy that people use involves images. Several of my interviewees found that a picture was able to convey something that could not be communicated with words and evoked a stronger reaction in people. In the European Union, pictures are now being printed on cigarette packages instead of written health warnings, indicating the same underlying belief that a picture is more powerful in conveying a message. The suggestion is not that a picture can communicate more than a written description, but that the picture evokes a more emotional response. Such an emotional reaction makes it easier for people to feel that others empathise with them. Thus a picture may enable empathy on both sides: the empathiser may find it easier to gain access to the experience, and because pictures more easily evoke emotions the person seeking empathy may feel more quickly understood. Nevertheless, it must be considered that personal distress caused, for example, by a picture may also disrupt an empathetic response. Reis (1998) describes how she reacted with distance to a situation that came emotionally too close. Some comments that are hurtful to people may possibly be explained by outsiders’ own struggles with the situation, as thinking about such a situation might confront them with their own vulnerabilities and fears.

In the case of early-onset dementia, and possibly other diseases, there is a barrier to reaching empathetic understanding, something that Moore and Gillespie (2014) have termed the ‘caregiving bind’. The authors argue that one reason why caregivers do not receive social recognition is because they conceal how much care is needed in order to protect the identity and reputation of the person for whom they are caring. Similarly, De Klerk (2012) found that parents may conceal the fact that their children have HIV/AIDS in order to protect the dignity of the family. In his classic book *The Cloak of Competence*, Edgerton (1993) describes how people with an intellectual disability try to conceal their disability and how caregivers not only help them in dealing with daily life, but also assist them in concealing their condition. Edgerton writes that no stigma is as basic as that for intellectual disability, in the sense that “a person so labelled is thought to be so completely lacking in basic competence” (1993:184). Just as with intellectual disability, dementia is highly stigmatised (Graham et al. 2003, in Milne 2010:227) and can lead to social death (Niehaus 2007, in de Klerk 2012:28; Kontos 2006; Ballenger 2006). Hellstrom and Torres (2013) found in their study that several couples expressed regret over having disclosed the diagnosis to others, as they noticed that their friends behaved differently afterwards. By emphasising how much care work is necessary for the person with dementia, family members risk stigmatisation. Although the stigma is mostly directed towards the person with the condition, it can also affect the whole family (Downs et al. 2013; Goffman 1963). As family members do not want their loved-one to be treated differently, they might conceal the care work that is necessary. This concealment is possible because care work is often invisible:

The kinds of work I have been discussing can be thought of as unacknowledged work in this sense; however, they are also literally invisible: much of the time they can’t be seen. … The work is noticeable when it is not completed … but cannot be seen when it is done (DeVault 1991:56, in Russell 2007:310).

As Buch argues, care practices are acts of social recognition, sustaining the personhood of the care receiver, preventing his or her social death (2013:641–642). Through caring, the effects of the illness are made invisible. Family members thus have to balance between wanting to protect the sick person and thus avoiding social death, and receiving empathetic understanding.

The above discussion shows that reaching empathetic understanding is a complex matter. Even though ‘a sorrow shared is a sorrow halved’, receiving understanding and empathy for one’s situation is not easily achieved. Moreover, when sharing something, one person might experience connection and the other estrangement.

In this article, I have described how family members of a person with early-onset dementia try to receive empathetic understanding from others. It is important to bear in mind that the interviews constitute a retrospective reflection on empathy. Feeling understood or misunderstood is not a fixed state but a dynamic process. People might feel understood regarding their situation with their family member with early-onset dementia in one moment, and then a comment will make them question whether this is really the case. The interviews I conducted, in which empathy was not a specific focus but appeared as a relevant topic, can be seen as offering a snapshot of this process.

## Conclusion and Implications

In this article, I have shown that empathic understanding is a complex matter that cannot be taken for granted. This article has illustrated the importance of taking the socio-cultural context into consideration when studying empathy. In a society, in which dementia is highly stigmatised, family members can feel inhibited to explain how much care they really provide. Similarly, in a society where independence and happiness are valued, chronically ill or disabled people might find it difficult to voice the experience of loss and dependence (Hoppe 2017; Watermeyer 2009). Thus not only interpersonal issues, but also the socio-cultural context shapes which aspect of an experience can more easily be shared and understood.

Another important contribution to the anthropological study of empathy is the acknowledgement that what needs to be understood is not simple. Instead of discussing whether empathetic understanding can be achieved, the multiplicity of an experience should be underlined. In a complex experience such as living with a person with early-onset dementia, various aspects can be understood, misunderstood or not understood. Based on the moment in time, but also on the relationship, people want different aspects to be understood. In some situation all that counts might be an emotional connection, whereas in others a more cognitive understanding of the situation is desired.

Different kinds of people might fulfil certain needs more easily than others. Moore and Gillespie suggest that health professionals are a “potentially unproblematic source of positive social recognition for caregivers” (2014:108) as the caregiving bind is less relevant. Moreover, as part of their profession, they are taught to not let their own issues disturb the understanding of the other. Halpern (2014:305) advises medical students to ask patients what the students are missing in their assessment, instead of saying that they know how the patient feels. Thus, professionals might be more inclined to have an attitude of curiosity. Yet, professionals seldom react with an emotional outburst to suffering. Also they often do not have the opportunity to participate in their patients’ world.

Friends or relatives might be more inclined to react emotionally to suffering. Moreover, they can more easily participate in the other person’s life and thereby gain an understanding of the situation. Although friends might also be curious about the situation of the other, they might rather look for similarities instead of reflecting on the differences of an experience. A wish to also share own experiences can further hinder empathetic listening. Furthermore, people’s own distress might disrupt an empathetic response, for example, if a relative has to come to terms with the fact that someone is seriously ill or if friends struggle with their own vulnerability or fear of death.

In the care of psychiatric patients and people with addictions in the Netherlands, for instance, ‘experience experts’ are used. Experience experts are people who share experiences with patients, for example they have had an addiction or have experienced psychiatric problems. With training, it is believed that they can provide different help than professionals who do not have these shared experiences (see Weerman 2013). Since these experience experts share patients’ emotional and psychological pain, it is assumed that they may be able to make a connection with them more easily, leading to a deeper understanding than is possible with a traditional therapist (Plooy 2007:18). Another possible source of support comes from support groups, which are seen as an important resource in helping people to better deal with their situation and decrease the burden they experience (Chen and Greenberg 2004:432).

Based on the empirical data of this article, however, it should not be assumed that an experience expert or members of a support group do necessarily understand the person in search of empathy better than a professional or someone who does not have such shared experiences. It first needs to be established whether the shared experiences are actually shared by both parties, and reflection is needed on the experiences that are not shared.

One important conclusion of this article is that people have different needs. Experience experts or support groups can fulfil certain needs, while a professional or friend can fulfil others. Regardless of the type of relationship, attuning to the needs of the person one wants to understand is crucial. Also the limits of empathy need to be acknowledged. “An empathic stance must include some notion and acceptance of the limits, failure, and even the impossibility of empathy” (Kirmayer 2008:470).

To conclude, having written an article on empathy based on relatively short encounters with people, I hope that I have conveyed what my interviewees wanted to convey.
